# Correlates of physical activity levels, muscle strength, working memory, and cognitive function in older adults

**DOI:** 10.3389/fnagi.2023.1283864

**Published:** 2023-12-14

**Authors:** Shufan Li, Peng Wang, Zhidong Cai, Wanting Jiang, Xin Xin, Xing Wang, Xiaojing Zhou

**Affiliations:** ^1^School of Physical Education, Shanghai University of Sport, Shanghai, China; ^2^Department of Physical Education, Suzhou University of Science and Technology, Suzhou, China; ^3^School of Sports and Health of Shanghai Lixin University of Accounting and Finance, Shanghai, China

**Keywords:** physical activity, muscle strength, working memory, cognitive function, older adults

## Abstract

**Objective:**

To explore the relationship between physical activity level, muscle strength, working memory and cognitive function in older adults.

**Methods:**

A cross-sectional research design was employed to recruit 120 older adults individuals aged 70 and above. Participants were asked to complete the International Physical Activity Questionnaire-Short Form and the Montreal Cognitive Assessment Scale. Data on variables such as grip strength and performance on the N-back task were collected. Data analysis involved the use of independent samples t-tests, χ2 tests, linear regression analysis, Pearson correlation analysis, and one-way analysis of variance (ANOVA).

**Results:**

The detection rate of cognitive dysfunction in older adults was 53.211%; 1-back correct rate had an explanatory power of 11.6% for the cognitive function scores of older adults (R^2^ = 0.116, *p* < 0.001); grip strength showed a significant positive correlation with 1-back correct rate (r = 0.417, *p* < 0.001), and was significantly correlated with the 0-back response time (r = −0.478), 1 -back response time (r = −0.441) were significantly negatively correlated (*p* < 0.001); physical activity level was significantly positively correlated with grip strength (r = 0.559, *p* < 0.001), and the difference in grip strength among older adults with different physical activity levels was statistically significant (*F* = 19.685, *p* < 0.001).

**Conclusion:**

Physical activity level, muscle strength, working memory, and cognitive function are closely related in older adults, and the relational pathway of physical activity → muscle strength → working memory → cognitive function may serve as a useful addition to promote the field of cognitive research in older adults. To identify and prevent cognitive decline in older adults, physical activity questionnaires, grip strength tests, and 1-back task tests can be extended to nursing homes and communities.

## Introduction

According to the World Health Organization (WHO), the number and proportion of people aged 60 and over in the population is increasing dramatically, and is expected to reach 2.1 billion by 2050, accounting for 22% of the world’s total population (Bajwa et al., 2019). With the aging of the population, preventing or delaying cognitive decline has become an urgent issue. Cognitive function refers to the ability to select, process, store, and extract information and to apply that information to guide one’s behavior (Jin, 2004). Cognitive dysfunction refers to multiple domains of cognitive functioning with varying degrees of impairment due to a variety of causes, the incidence of which increases with age and can reach 33.59% (He Shuning et al., 2023). Alzheimer’s disease has become the third leading cause of threat to human life and health after cardiovascular diseases and malignant tumors (Liu Jin et al., 2017).

Cognitive decline in the older adults is first manifested through impairment in working memory (D'esposito and Postle, 2015). Working memory refers to the temporarily limited system that individuals use to store and process information during cognitive tasks. It is considered a decisive process for reasoning, decision-making, and behavior and is regarded as the core of cognitive activities (Baddeley, 1992; Elliott et al., 2011). Working memory declines with age and significantly decreases after the age of 60 (Wang et al., 2011). The prefrontal cortex decline theory suggests a close relationship between brain aging and prefrontal cortex degeneration, with the prefrontal cortex being a crucial brain region involved in working memory (McSween et al., 2019). Therefore, preventing and delaying cognitive decline in the older adults should focus on their working memory.

Exercise can effectively prevent or delay cognitive decline in the older adults, particularly in working memory. The brain exhibits plasticity (Gutchess, 2014), and exercise can lead to physiological and metabolic changes in the body, promoting the maintenance of brain structure and function (Raffin et al., 2023), thus enhancing cognitive function. It serves as a safe and effective alternative for delaying the decline in working memory (McSween et al., 2019). Studies have found that older adults individuals engaged in regular physical activity with higher levels of physical activity and stronger muscle strength have a reduced risk of cognitive decline by 30 to 46% (Angevaren et al., 2010; Northey et al., 2018; Seo and Lee, 2022). Resistance training can maintain and enhance muscle strength in the older adults, and higher muscle strength is associated with better performance in working memory (Fontes et al., 2017; Firth et al., 2018).

Previous studies have examined the relationship between physical activity level, muscle strength, working memory, and cognitive functioning, finding that physical activity level is positively correlated with muscle strength (Sánchez-Sánchez et al., 2019). Muscle strength is a unidirectional predictor of working memory (Firth et al., 2018). Working memory is one of the important dimensions of cognitive function. By analyzing the previous studies, we found that the following questions still need to be addressed in this field: Is there a difference in muscle strength among older adults with different levels of physical activity? Does working memory differ among older adults of different genders and muscle strengths? The N-back task can be used as a measurement tool for working memory, so which loaded task reflects working memory more sensitively and accurately? To what extent does working memory explain overall cognitive functioning? Using a cross-sectional research design, this study intends to explore the relationship between the four variables, and also to clarify whether there is a relational pathway of physical activity → muscle strength → working memory → cognitive function, in order to provide evidence for preventing or delaying cognitive decline in the older adults and offer clinical references.

## Research subjects and methods

### Research subjects

In four older adults service centers in Shanghai, 120 individuals aged 70 and above were recruited through methods such as health education lectures and posting recruitment posters, based on the principle of voluntariness. Inclusion criteria: (1) Individuals aged 70 and above; (2) Right-handed; (3) Good physical condition; (4) Absence of severe cardiovascular diseases; (5) Normal vision and hearing; (6) Normal mental state, able to communicate verbally, and willing to cooperate in completing the survey; (7) Willing to sign the informed consent form. Exclusion criteria: (1) Severe cardiovascular diseases or major organic diseases; (2) Severe muscular diseases, inability to stand for prolonged periods; (3) Presence of contraindications to exercise; (4) Poor vision or hearing, unable to complete the tests; (5) Long-term or recent use of psychotropic drugs, medications affecting physical activity, cholinergic inhibitors, and other relevant medications.

**Figure 1:**
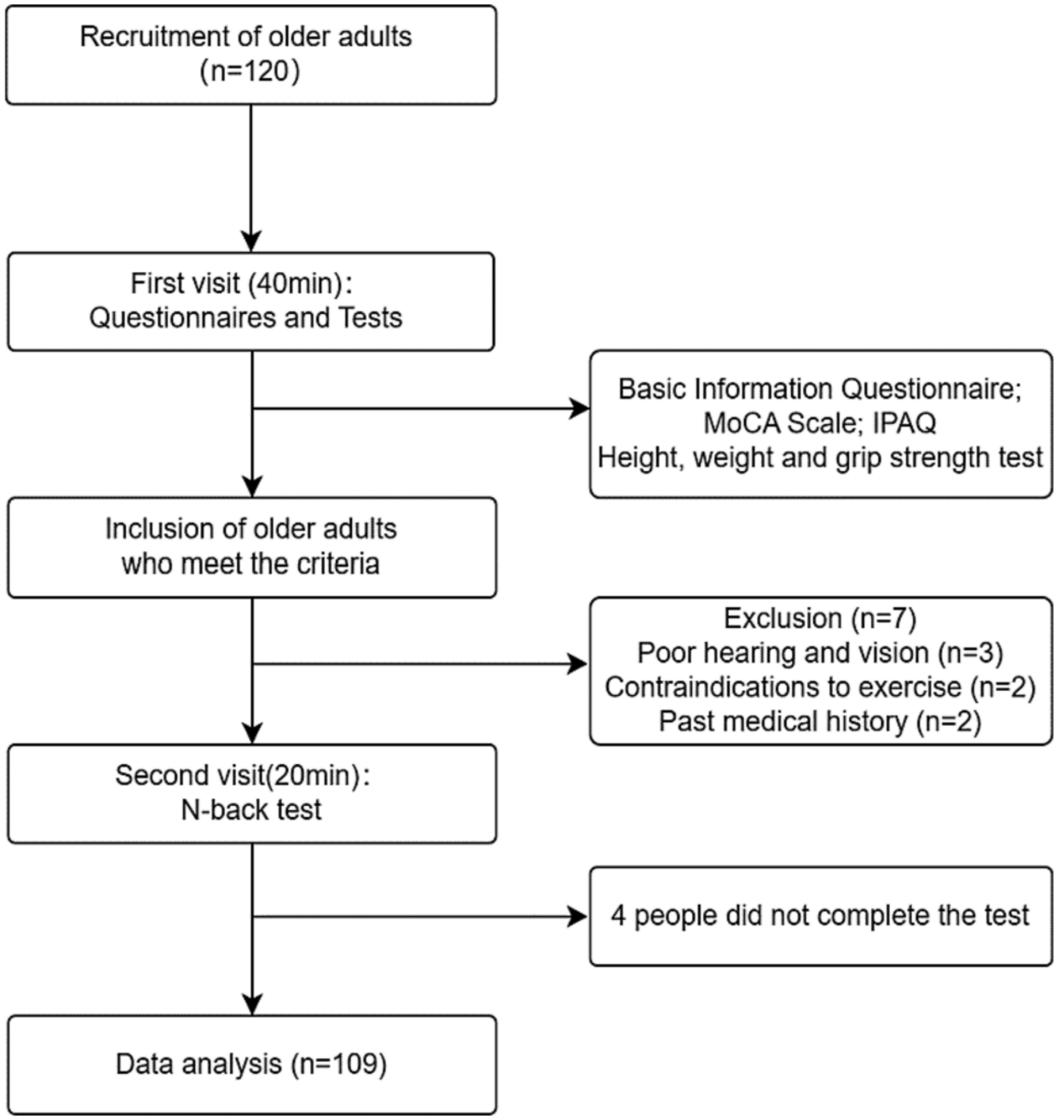
Testing flowchart.

All tests were conducted between 13:30 and 16:30. Participants visited the laboratory twice. During the first visit, the testing procedure was explained, informed consent was obtained, and participants filled out a questionnaire on basic information, the Montreal Cognitive Assessment (MoCA), and the International Physical Activity Questionnaire (IPAQ). Additionally, height, weight, and grip strength tests were performed, taking a total of 40 minutes. On the second visit, a working memory task test was conducted, lasting 20 min. The testing protocol is illustrated in Figure 1. Participants refrained from vigorous exercise 24 h before the test and abstained from consuming caffeinated or alcoholic beverages. All participants volunteered for the experiment, signed informed consent forms, and the study adhered to the latest version of the Helsinki Declaration ethical requirements. Ethical approval was obtained from the Ethics Committee of Shanghai University of Sport (102772020RT060).

### Testing tools

#### Basic information questionnaire

The basic information survey questionnaire includes: name, age, gender, marital status, source of income, economic status, handedness, presence of contraindications to exercise, medical history, and medication usage.

#### Montreal cognitive assessment scale

The scale is widely used to assess cognitive functioning in older adults and consists of 8 domains: visuospatial/executive functioning, naming, memory, attention, verbal fluency, abstract thinking, delayed recall, and orientation, with a total score of 30 points, with the higher the score the better the cognitive functioning. The cut-off value for identifying cognitive normalcy and cognitive dysfunction was <26 points. To avoid the influence of years of education, 2 points were added to the total score of the scale for ≤6 years of education, 1 point was added for >6 years and < 12 years of education, and no points were added for >12 years of education, and no points were added if the total score was more than 30 after the addition. The MoCA Beijing version was chosen for this study, with a retest reliability of 0.857 (Zhang Lixiu, 2007).

#### International physical activity questionnaire short form

The questionnaire consisted of 7 questions, 6 of which asked individuals about their physical activity, including high intensity physical activity, moderate intensity physical activity and walking, and asked about the 1-week frequency and cumulative time per day of the different intensity activities, and the weekly physical activity level of the study participants was calculated and divided into high and medium-low groups according to the relevant criteria, and the higher the group, the higher the daily physical activity of the individual, and the International Physical Activity Questionnaire-Short Form of the retest reliability coefficient was 0.718 (Qu Ningning, 2004).

#### Height, weight, and grip strength tests

Height was measured using a stadiometer. Participants stood barefoot, facing away from the stadiometer, with their back against the measurement board, maintaining a natural posture and straight head, and looking straight ahead. Measurements were recorded in centimeters, rounded to one decimal place. Weight was measured using a scale. Participants stood barefoot, naturally positioned in the center of the scale, maintaining stability. Measurements were recorded in kilograms, rounded to one decimal place. Height and weight were measured twice, and the average values were calculated. Body Mass Index (BMI) was calculated using the formula BMI = weight (in kilograms) divided by the square of height (in meters).

Grip strength can reflect the body’s overall muscle strength and physical function, with a high degree of practicality and sensitivity (Ma Xin et al., 2021). The subject is in a standing position with the arms straight and hanging down at the side of the body. Holding a grip strength meter in their hand, they squeezed the handle with maximum force for 3 to 5 s, and the left and right hands were tested three times, with a 30-s interval between tests, and the average value was taken as the grip strength value. Low muscle strength is defined as a grip strength of <28 kg for men and < 18 kg for women (Chen et al., 2014).

#### N-back task

The assessment of working memory utilized the N-back task paradigm, a classic experimental paradigm in psychology. Which was programmed using the psychological experiment’s programming system platform E-prime 2.0, and the stimulus types were numbers, and the subjects responded to the stimulus key presses on the computer according to the requirements of the N-back task, and their response time and correct rate were recorded. The N-back task of this test was designed with three kinds of cognitive loads, including 0-back, 1-back, and 2-back, and the 0-back task required subjects to compare the current number with “0” and press “1” for the same number, and press “2” for the different number. The 1-back task requires subjects to start from the second number and determine whether the current number is the same as the previous number, pressing 1 for the same number and 2 for different numbers. 2-back task requires subjects to start from the second number and determine whether the current number is the same as the previous number, pressing 1 for the same number and 2 for different numbers. “The 2-back task required subjects to start from the third digit and determine whether the current digit was the same as the previous digit, pressing “1″ for the same digit and “2″ for different digits. The instructions and exercises were set up before the start of the formal experiment, and each cognitive load task was repeated five times. At the beginning of the experiment, a “+” appeared for 500 ms to maintain the subjects’ attention, and then 10 numbers between 0 and 9 were randomly presented, with a number presentation time of 500 ms, followed by an empty screen of 2000 ms, during which the next number stimulus was automatically presented if no response was made. At the end of each cognitive load task, a prompt “Rest 30s” appeared. The test lasted approximately 15 min.

### Mathematical statistics

Statistical analysis was conducted using SPSS version 26.0. Descriptive statistics for continuous data were presented as mean ± standard deviation, with results rounded to three decimal places. Group comparisons for continuous data were performed using independent samples t-tests. Descriptions for categorical data were presented as frequencies (n), and group comparisons were conducted using χ2 tests. Linear regression analysis was employed to explore the explanatory power of working memory on cognitive function. Pearson correlation analysis was used to examine the relationships between muscle strength and working memory, as well as between physical activity and muscle strength. One-way analysis of variance (ANOVA) was used to investigate the differences in working memory among older adults with different genders and muscle strengths; one-way ANOVA and LSD *post hoc* multiple tests were used to compare the differences in grip strength among older adults with different levels of physical activity. All statistical inferences were two-tailed, with a significance level set at α = 0.05, indicating statistically significant differences.

## Research results

### Differences in physical activity levels, muscle strength, and working memory in older adults with different levels of cognitive functioning

109 older adults participated in the study with a mean age of 80.101 ± 6.519 and a detection rate of 53.211% for cognitive dysfunction. The differences in years of education, physical activity level, grip strength, 0-back response time, 1-back response time, and 1-back correct rate were statistically significant (all *p* < 0.05), and the differences were not statistically significant (all *p* > 0.05) on the other variables when normal older adults were compared to cognitively dysfunctional older adults, as shown in Table 1.

**Table 1:** Comparison of differences in physical activity levels, muscle strength and working memory between normal and cognitively impaired older adults.

Variables	Cognitive functioning score			Comparison among groups
	Overall(109)	Cognitive dysfunction(58)	Normal(51)	
Age/years	80.101 ± 6.519	80.500 ± 6.239	79.647 ± 6.858	t = 0.680, *p* = 0.498
Height (m)	1.596 ± 0.072	1.586 ± 0.073	1.609 ± 0.070	t = −1.692, *p* = 0.094
Weight (kg)	58.014 ± 10.849	56.871 ± 10.115	59.367 ± 11.618	t = −1.188, *p* = 0.237
BMI(kg/m2)	22.744 ± 3.443	22.565 ± 3.452	22.956 ± 3.455	t = −0.584, *p* = 0.561
Gender				χ2 = 1.457, *p* = 0.227
Male	51	24	27	
Female	58	34	24	
Marital Status				χ2 = 0.715, *p* = 0.398
Married	53	26	27	
Single, Divorced, or Widowed	56	32	24	
Source of Income				χ2 = 0.572, *p* = 0.751
Retirement Pension	41	22	19	
Gifts from Children and Other Relatives	48	24	24	
Other	20	12	8	
Economic Status				χ2 = 5.318, *p* = 0.070
Good	16	6	10	
Average	72	44	28	
Poor	21	8	13	
Years of education (years)	11.284 ± 3.892	10.500 ± 4.062	12.176 ± 3.520	t = −2.287, *p* = 0.024
Physical activity (MET-min/week)	1568.142 ± 1176.872	1261.405 ± 902.784	1916.980 ± 1352.508	t = −2.934, *p* = 0.004
Grip strength (kg)	24.757 ± 8.283	22.093 ± 6.444	27.786 ± 9.125	t = −3.715, *P*<0.001
0-back response time	643.298 ± 181.886	701.589 ± 213.777	577.006 ± 104.572	t = 3.935, *P*<0.001
1-back response time	775.757 ± 223.204	839.382 ± 262.136	703.399 ± 138.705	t = 3.441, *p* = 0.001
2-back response time	958.898 ± 209.707	975.115 ± 230.335	940.456 ± 184.002	t = 0.860, *p* = 0.392
0-back correct rate	0.927 ± 0.102	0.924 ± 0.125	0.930 ± 0.067	t = −0.289, *p* = 0.773
1-back correct rate	0.791 ± 0.175	0.751 ± 0.184	0.836 ± 0.153	t = −2.635, *p* = 0.010
2-back correct rate	0.701 ± 0.172	0.675 ± 0.155	0.731 ± 0.187	t = −1.702, *p* = 0.092

### The extent To which working memory tasks explain overall cognitive functioning

To examine the extent to which working memory explains MoCA scores, multiple linear regression analyses (stepwise method) were conducted with MoCA scores as the dependent variable and 0-back response time, 1-back response time, 2-back response time, 0-back correct rate, 1-back correct rate, and 2-back correct rate as the independent variables, and the results showed that the regression model passed the significance test (*F* = 14.001, *p* < 0.001, R^2^ = 0.116). 1-back correct rate (Beta = 0.340, *p* < 0.001) was predictive of MoCA scores in older adults, while the other indicators were excluded. With tolerances >0.1 for each variable and VIF values <5, multicollinearity can be largely excluded from the results of this study. 1-back correct rate had 11.6% explanatory power for cognitive function scores. See Table 2 for details.

**Table 2:** Multiple linear regression analyses of working memory tasks explaining overall.

Independent variables	B	95%CI		Beta	Coefficient significance test		SE	Collinearity diagnostics	
		lower limit	upper limit		*t*-value	*p*-value		Tolerance	VIF
1-back correct rate	9.496	4.465	14.527	0.340	3.742	<0.001	2.538	1.000	1.000

Model Summary F = 14.001***, R = 0.340, R^2^ = 0.116, Adjusted R^2^ = 0.107.

*** indicates *p* < 0.001.

### Relationship between muscle strength and working memory

**Figure 2:**
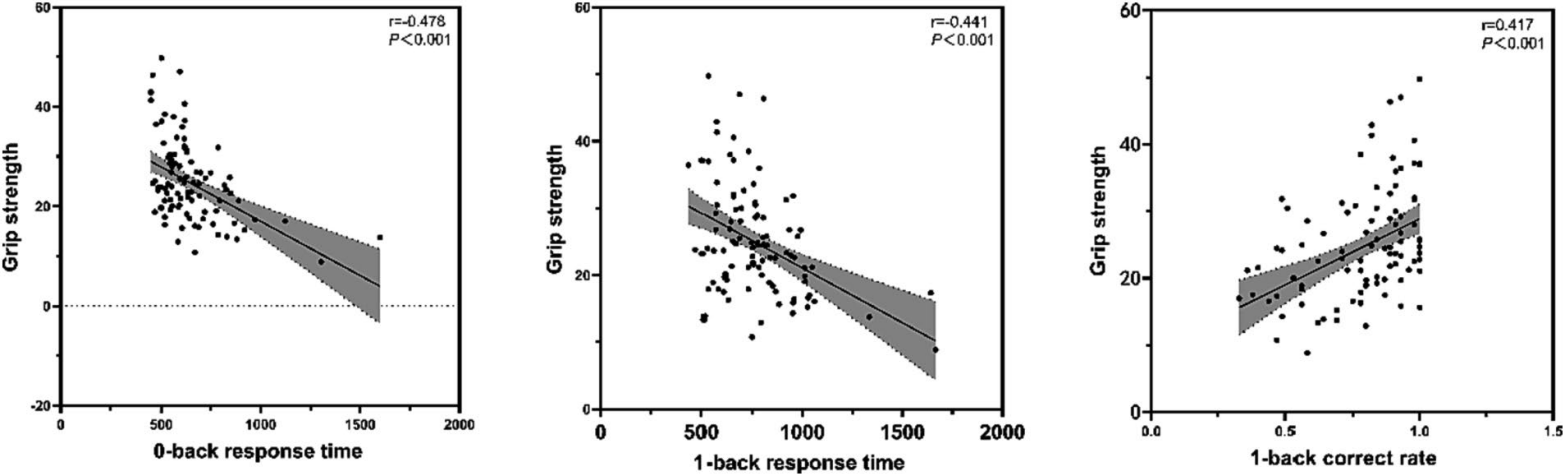
Relationship between muscle strength and working memory in older adults.

There were significant differences between normal and cognitively dysfunctional older adults in 0-back response time, 1-back response time, and 1-back correct rate. Pearson correlation coefficients were used to examine the relationship between grip strength and the above working memory indices (Figure 2). The results showed that grip strength was significantly and positively correlated with 1-back correct rate (r = 0.417, *p* < 0.001), and significantly and negatively correlated with 0-back response time (r = −0.478) and 1-back response time (r = −0.441) (*p* < 0.001).

As shown in Table 3, the older adults of different genders were divided into normal muscle strength group and low muscle strength group, and the differences among the four groups on the working memory task were statistically significant (all *p* < 0.01). The different muscle strength male group was better than the different muscle strength female group on all variables, and the low muscle strength male group was better than the normal muscle strength female group on 1-back response time and 1-back correct rate index.

**Table 3:** Comparison of differences in working memory among older adults of different sexes and muscle strengths.

Variables	Muscle strength (female)		Muscle strength (male)		*F*-value	*p*-value
	Normal (38)	Low (20)	Normal (31)	Low (20)		
0-back response time	618.416 ± 114.667	851.143 ± 292.131	560.231 ± 73.232	611.483 ± 97.976	16.016	<0.001
1-back response time	780.856 ± 146.467	987.310 ± 355.887	665.855 ± 116.547	724.865 ± 153.989	11.404	<0.001
1-back correct rate	0.808 ± 0.165	0.627 ± 0.162	0.869 ± 0.127	0.802 ± 0.173	10.153	<0.001

### Relationship between physical activity level and muscle strength

**Figure 3:**
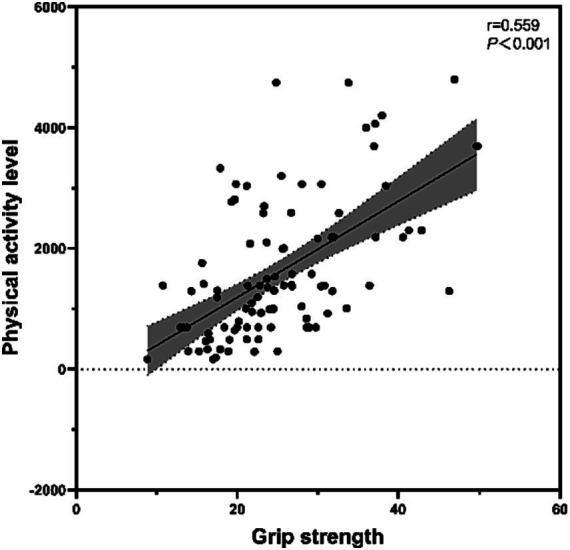
Relationship between physical activity level and muscle strength.

Pearson’s correlation coefficient was used to examine the relationship between physical activity level and muscle strength (Figure 3), and physical activity level was significantly and positively correlated with grip strength (r = 0.559, *p* < 0.001). The mean grip strength of older adults with high physical activity level (n = 17) was 31.495 ± 9.195, that of older adults with medium physical activity level (n = 69) was 25.501 ± 7.399, and that of older adults with low physical activity level (n = 23) was 17.543 ± 3.858, and the difference between older adults with different levels of physical activity was statistically significant in terms of grip strength (*F* = 19.685, *p* < 0.001). *Post hoc* multiple comparisons found that there was a significant difference in grip strength between older adults with high and moderate physical activity levels and older adults with low physical activity (*p* < 0.01).

## Discussion

The results of this study indicate that the higher the physical activity level of older adults, the greater their muscle strength, which is generally consistent with previous findings. There is a positive correlation between physical activity level and muscle strength (Papiol et al., 2016; Ramsey et al., 2021; Seo and Lee, 2022), with the level of physical activity being an important influencing factor for muscle hypertrophy and increased muscle strength (Adelnia et al., 2019). Possible reasons for this are: exercise induces the production of adenosine triphosphate in skeletal muscle mitochondria, which improves aerobic capacity and promotes muscle protein synthesis (Erlich et al., 2016). It also affects the expression of muscle growth inhibitors and autophagy proteins mRNA (messenger RNA) (Yan et al., 2012; Ko et al., 2014). which is an important strategy for preventing muscle atrophy and increasing muscle strength (Sousa et al., 2017).

This study further confirms the correlation between muscle strength and working memory. In older adults, greater muscle strength is associated with faster response times and higher accuracy in completing working memory tasks (Filardi et al., 2022). Possible reasons are: the prefrontal lobe is an important brain region for working memory, and its activation level is closely related to working memory (D'esposito and Postle, 2015). Muscle strength is positively correlated with whole brain volume, white matter volume, and gray matter volume in the right temporal pole and bilateral anterior medial ventral tegmentum (Kilgour et al., 2014; Dercon et al., 2021). Increased muscle strength positively affects working memory by increasing activation levels in specific areas of the prefrontal lobe (Kilgour et al., 2014; Kobori et al., 2015; Cai et al., 2023). It is noteworthy that muscle strength has gender differences, which may be influenced by physiological differences between males and females, hormonal changes, and aging mechanisms (Baumgartner et al., 1999).

The present study found that working memory positively affects overall cognitive functioning, and that the rate of correctly completing a 1-back task is predictive of it. Working memory is an important component of cognitive function and is the first cognitive function to be impaired in neurodegenerative diseases such as Alzheimer’s disease (D'esposito and Postle, 2015). From the perspective of working memory load, the 0-back task belongs to low memory load, reflecting information recognition and maintenance of working memory; the 1-back task belongs to medium memory load, reflecting information recognition, maintenance, and updating of working memory; the 2-back task belongs to high memory load, reflecting information recognition, maintenance, updating, and inhibition of working memory (Liu Cong et al., 2017). The mean age of the older adults included in this study was 80.101 ± 6.519, and this population has cognitive decline, for which the 1-back task is quite difficult, and the 2-back task may have exceeded their cognitive abilities. Therefore, completion of the 1-back task can be used as a sensitive and accurate measure of working memory in older adults.

The present study identified the relational path of physical activity → muscle strength → working memory → cognitive function from the exploration of the above relationships, which may be a useful addition to promote the field of cognitive research in older adults. In order to identify and prevent cognitive decline in older adults, the Physical Activity Questionnaire, the Grip Strength Test, and the 1-Back Task Test can be extended to nursing homes and communities. Since no previous studies have explored the relationship between the four, the mechanism of their influence is unclear, and more high-quality studies are needed to confirm this relational pathway in the future.

The present study has the following limitations: physical activity levels were derived from subjective reports, which may have some bias, and the use of objective measurement tools is recommended for the future; as a cross-sectional study, it still needs to be further confirmed by longitudinal studies; and this paper has not yet explored the influence of other factors such as mental health status, which should be emphasized in future studies.

## Conclusion

Physical activity level, muscle strength, working memory, and cognitive function are closely related in older adults, and the relational pathway of physical activity → muscle strength → working memory → cognitive function may serve as a useful addition to promote the field of cognitive research in older adults. To identify and prevent cognitive decline in older adults, physical activity questionnaires, grip strength tests, and 1-back task tests can be extended to nursing homes and communities.

## Data availability statement

The original contributions presented in the study are included in the article/supplementary material, further inquiries can be directed to the corresponding author.

## Ethics statement

The studies involving humans were approved by Ethics Committee of the Shanghai University of Sport (102772020RT060). The studies were conducted in accordance with the local legislation and institutional requirements. The participants provided their written informed consent to participate in this study.

## Author contributions

SL: Conceptualization, Writing – original draft, Data curation, Investigation, Methodology. PW: Data curation, Methodology, Writing – original draft. ZC: Data curation, Investigation, Writing – original draft. WJ: Investigation, Writing – original draft. XX: Investigation, Writing – original draft. XW: Investigation, Funding acquisition, Writing – original draft. XZ: Writing – review & editing.
